# Interactive effects of a common *γ-glutamyltransferase 1* variant and low high-density lipoprotein-cholesterol on diabetic macro- and micro-angiopathy

**DOI:** 10.1186/s12933-015-0212-5

**Published:** 2015-05-08

**Authors:** Hideaki Jinnouchi, Kazunori Morita, Takahiro Tanaka, Ayami Kajiwara, Yuki Kawata, Kentaro Oniki, Junji Saruwatari, Kazuko Nakagawa, Koji Otake, Yasuhiro Ogata, Akira Yoshida, Seiji Hokimoto, Hisao Ogawa

**Affiliations:** Division of Preventive Cardiology, Department of Cardiovascular Medicine, Kumamoto University Hospital, Kumamoto, Japan; Jinnouchi Clinic, Diabetes Care Center, Kumamoto, Japan; Division of Pharmacology and Therapeutics, Graduate School of Pharmaceutical Sciences, Kumamoto University, 5-1, Oe-honmachi, 862-0973 Kumamoto, Japan; Center for Clinical Pharmaceutical Sciences, Kumamoto University, Kumamoto, Japan; Japanese Red Cross Kumamoto Health Care Center, Kumamoto, Japan; Department of Cardiovascular Medicine, Faculty of Life Sciences, Graduate School of Medical Sciences, Kumamoto University, Kumamoto, Japan

**Keywords:** Brachial-Ankle Pulse Wave Velocity, Diabetic Angiopathies, Diabetic Retinopathy, γ-Glutamyltransferase, High Density Lipoprotein Cholesterol, Oxidative Stress, Single Nucleotide Polymorphism, Type 2 Diabetes Mellitus

## Abstract

**Background:**

We investigated the clinical relevance of a common variant, rs4820599, in the γ*-glutamyltransferase (GGT)1* gene, associated with the serum GGT level, in Japanese type 2 diabetes mellitus (T2DM) subjects.

**Methods:**

We conducted a retrospective longitudinal study (4.9 ± 2.5 years) including 352 T2DM patients (T2DM subjects) and a cross-sectional study including 796 health screening program participants (general subjects). A real-time TaqMan allelic discrimination assay was used to identify the genotypes. Risk factors for a high brachial-ankle pulse wave velocity (baPWV) (≥1750 cm/sec) or diabetic retinopathy (DR) were determined using a generalized estimating equations approach, receiver operating characteristic (ROC) analysis or Cox proportional hazards model, *etc.*

**Results:**

The frequency of the *GGT1* G allele was 20.8% in the T2DM subjects, and no associations were found between the *GGT1* genotype and risk of T2DM. The mean log GGT values in the T2DM and general subjects were significantly higher among G allele carriers than non-carriers. The G allele and a low HDL-C level were identified to be risk factors for a high baPWV in the T2DM subjects [odds ratio (OR) 1.80, *P* = 0.008; OR 1.71, *P* = 0.03; respectively), and a significant interactive effect between these factors was found on the risk of a high baPWV and DR. The HDL-C level at baseline was a significant predictor of a high baPWV only in G allele carriers according to the ROC analysis. This result regarding baPWV in the T2DM subjects was replicated in the general population. Meanwhile, the *GGT1* genotype was not associated with the risk of DR, although it affected the principal factors involved in the risk of DR, and a low HDL-C level was also found to be a risk factor for DR only in G allele carriers.

**Conclusions:**

We herein describe for the first time the significant interactive effects of the *GGT1* G allele and a low HDL-C level on a high baPWV and DR. These findings may encourage future clinical trials comparing the efficacy of agents increasing the HDL-C levels among the *GGT1* genotypes. However, well-designed studies in larger cohorts are needed to confirm our results.

**Electronic supplementary material:**

The online version of this article (doi:10.1186/s12933-015-0212-5) contains supplementary material, which is available to authorized users.

## Introduction

The level of serum γ-glutamyltransferase (GGT), a widely used index for excessive alcohol consumption and/or liver dysfunction, has been found to be associated with an increased risk of diabetes, metabolic syndrome and cardiovascular disease (CVD), including hypertension, coronary heart disease (CHD) and heart failure (HF), as well as both cardiovascular and all-cause mortality [1-5]. The pro-oxidant effect of GGT is proposed to be the biological mechanism linking GGT to various cardiovascular events, as a strong GGT activity and the GGT-mediated oxidation of low-density lipoprotein cholesterol (LDL-C) have been identified in the core tissue of atheromas, likely influencing plaque evolution and rupture [6-8]. Most serum GGT is bound to lipoproteins, such as anti-atherogenic apolipoprotein (Apo) A-I, contained in high-density lipoprotein cholesterol (HDL-C), and atherogenic Apo B [6,8-11]. Huseby [10] reported that 70% of the total serum GGT activity is detected in association with HDL-C.

Classic ‘diabetic dyslipidemia’ is characterized by the so-called atherogenic lipid triad, consisting of increased levels of small dense LDL particles and triglyceride-rich lipoproteins and a decreased level of HDL-C, associated with an increase in non-HDL cholesterol [12-16]. After the introduction of statins, clinical emphasis first focused on LDL-C lowering as the prime lipid target [12]. However, treated patients remained at high risk of cardiovascular events despite receiving the best standards of care, including high-dose statins [12-14,16,17]. Under such conditions, a raised triglyceride concentration is strongly associated with a low HDL-C level. Hence, the Residual Risk Reduction Initiative defines atherogenic dyslipidemia as the imbalance between Apo B and Apo A-I containing lipoproteins and identified this factor to be a key contributor to residual cardiovascular risks [17]. Next, the effect of potential HDL-C raising dominated research, with less focus on triglycerides [12,13,15-17]. In the Action to Control Cardiovascular Risk in Diabetes (ACCORD) trial, the use of fenofibrate in patients with T2DM receiving simvastatin who were at high risk of cardiovascular disease greatly reduced the triglyceride levels, although it hardly increased the HDL-C levels and did not reduce cardiovascular risks [14]. Nevertheless, the results of this trial suggest that fenofibrate reduces the risk of CHD in patients with high levels of triglycerides and low levels of HDL-C [14]. In 2014, a large meta-analysis of randomized controlled trials of agents that increase the HDL-C level, *i.e.* niacin, fibrates and cholesteryl ester transfer protein inhibitors, reported that none of these drugs reduce cardiovascular events in patients treated with statins [18]. Additionally, a recent study using an analytical approach that accounts for the potential pleiotropic effects of single-nucleotide polymorphisms (SNPs) on the triglyceride, LDL-C and HDL-C levels and risk of CHD simultaneously in a large sample demonstrated that plasma triglyceride-rich lipoproteins causally influence the risk of CHD rather than low HDL-C [19]. Consequently, the classic HDL hypothesis, defined as the concept that interventions to raise the HDL-C concentration will reduce cardiovascular risks, is gradually being replaced by the triglyceride hypothesis and HDL function hypothesis [12-14,20-22]. On the other hand, in patients with diabetic microvascular complications (MVCs), a large prospective study of newly diagnosed T2DM subjects showed that a 0.03 mmol/L increase in HDL-C is associated with a 1% decrease in the MVC risk and that patients achieving the HDL-C goals (>1.0 mmol/L for males and 1.25 mmol/L for females) have a 11% lower risk of MVC versus non-achievers [23]. The ACCORD trial also demonstrated a large reduction in the progression of diabetic retinopathy (DR) in patients with T2DM [24]. Furthermore, Du et al. [25] identified extravasated Apo A-I and Apo B in human diabetic eyes and revealed that native HDL completely blocks oxidative stress and the apoptosis of retinal pigment epithelial cells induced by heavily oxidized glycated LDL, thereby suggesting an important new role for extravasated and modified plasma lipoproteins in promoting DR.

The serum level of GGT is influenced by both environmental and genetic factors, and its heritability has been estimated to be 52% [26]. Furthermore, genome-wide association studies (GWAS) have identified an association between the *GGT1* gene loci and the GGT level in different ethnic groups [27-29]. However, the association of these loci with diabetes and/or CVD has not yet been reported. We herein present for the first time the significant interactive effects of a *GGT1* variant, rs4820599, and low HDL-C on a high pulse wave velocity (PWV), a surrogate marker of arterial stiffness, and DR in Japanese subjects with type 2 diabetes mellitus (T2DM).

## Methods

We conducted a retrospective longitudinal study including 352 patients with T2DM (231 males and 121 females, 60.0 ± 11.0 years: T2DM subjects) who had been treated at the Jinnouchi Clinic, Diabetes Care Center for more than six months between February 2002 and January 2011 and a cross-sectional study including 796 subjects (517 males and 279 females, 62.1 ± 12.4 years: general subjects) who had participated in a health screening program at the Japanese Red Cross Kumamoto Hospital Health Center. Subjects with a positive serological test for hepatitis viruses or hepatobiliary diseases other than fatty liver disease were excluded, whereas those with a history of CHD, 37 cases (10.5%) among the T2DM subjects and 37 cases (4.6%) among the general subjects, were included. The study protocol was approved by the institutional ethics committees, and written informed consent was obtained from each subject. The study was performed in accordance with the Declaration of Helsinki.

Height and weight were measured using standard protocols, and the body mass index (BMI) was calculated. Blood pressure (BP) was measured after the subjects rested in the sitting position. Hypertension was defined as a systolic BP of ≥ 140 mmHg, diastolic BP of ≥ 90 mmHg or history of hypertension. The brachial-ankle PWV (baPWV) was measured using an automated waveform analyzer (Omron-Colin, Tokyo, Japan). Pneumatic cuffs were wrapped around both upper arms and ankles and connected to a plethysmographic sensor to determine the volume pulse waveform. The highest value of baPWV measured on either side of each patient was used for the analysis. The value of baPWV at each follow-up visit was dichotomized by 1,750 cm/sec, which has been reported as the cut-off value for predicting the onset of stroke or cardiovascular disease [30].

Fatty liver was diagnosed based on the following four criteria: a diffuse hyperechoic echo texture (bright liver), increased echo texture in comparison to the kidneys, vascular blurring and deep attenuation [31]. DR was diagnosed by a professional ophthalmologist using direct ophthalmoscopy or fundus fluorescein angiography. DR was staged as no retinopathy, nonproliferative DR (NPDR) or proliferative DR (PDR) according to the criteria determined at the third national ophthalmology conference held in 1985. The occurrence of DR was defined as having no sign of DR signs in both eyes at baseline with the development of NPDR or PDR in either eye during the follow-up period.

Laboratory tests were performed using the standard methods of the Japan Society of Clinical Chemistry. Dyslipidemia was defined as a triglyceride level of ≥ 1.7 mmol/L, HDL-C level of < 1.0 mmol/L, LDL-C level of ≥ 3.6 mmol/L or history of dyslipidemia. Diabetes was defined as a fasting plasma glucose level of ≥ 7.0 mmol/L or casual or two-hour glucose level of ≥ 11.1 mmol/L after a 75 g oral glucose load test and a hemoglobin A1c (HbA1c) level of ≥ 6.5% or history of diabetes. Patients with type 1 diabetes were excluded based on their clinical manifestations. Information regarding smoking habits and alcohol intake was obtained via face-to-face interviews with health care providers.

Genomic DNA was isolated from EDTA-preserved blood samples using an automated DNA isolation system (NA-3000) (Kurabo, Osaka, Japan). A real-time TaqMan allelic discrimination assay was used to identify the *GGT1* (rs4820599, assay ID: C_2446754_10) and *aldehyde dehydrogenase 2 (ALDH2)* (rs671, assay ID: C_11703892_10) polymorphisms, and genotyping was performed according to the manufacturer’s protocol (Applied Biosystems, Tokyo, Japan). To ensure the quality of the genotyping, we included DNA samples as internal controls, with hidden samples of known genotypes and negative controls (water).

The data are presented as the mean ± standard deviation or proportion for categorical variables. Student’s *t*-test or a one-way analysis of variance and Fisher's exact test were used for comparisons of continuous and categorical variables, respectively. Factors influencing the GGT level were determined using a univariate linear regression analysis. Independent models were used for the analysis of the risk of a high baPWV (≥1,750 cm/sec), with calculation of the odds ratio (OR) and 95% confidence interval (95%CI) as well as the analysis of the baPWV values obtained during the observation period, with calculation of the adjusted partial regression coefficient (Β). We developed multiple regression models by incorporating potentially confounding factors that showed a *P* value of < 0.10 in the univariate models. Receiver operating characteristic (ROC) curves were determined to evaluate the predictive performance of these factors for detecting a high baPWV, with calculation of the area under the curve (AUC). We also determined the cut-off values for these factors as the point with the shortest distance from the left upper corner of the graph. Multivariate-adjusted hazard ratios (HRs) and 95%CIs for the cumulative incidence of the development of DR were examined using a Cox proportional hazards model. In addition, the interactive effects of the *GGT1* genotypes and potentially confounding factors on the baPWV or incidence of DR were assessed. A *P* value of < 0.05 was considered to be statistically significant. All statistical analyses were performed using the SPSS software package (version 17.0, IBM Japan Inc., Tokyo, Japan).

## Results

The frequency of the *GGT1* G allele was 20.8% and 22.1% in the T2DM patients and general subjects, respectively. The observed genotype frequency distributions in both populations were consistent with the Hardy-Weinberg equilibrium. No associations were found between the *GGT1* genotype and the risk of T2DM.

The mean follow-up duration in the T2DM subjects was 4.9 ± 2.5 years. The clinical characteristics of the T2DM subjects at baseline stratified by the *GGT1* genotype are shown in Table 1. The *GGT1* genotypes were not significantly associated with any of the parameters. However, the mean log GGT value was significantly higher in *GGT1* G allele carriers than in non-carriers (1.52 ± 0.30 IU/L vs. 1.45 ± 0.30 IU/L, *P* = 0.04). The other parameters did not differ between G allele carriers and non-carriers.

**Table 1:** **Clinical characteristics of T2DM stratified by the** ***GGT1*** **genotype at baseline**

	**A/A (n = 219)**	**A/G (n = 120)**	**G/G (n = 13)**	***P*** **-value**
Female (%)	33.3	35.0	46.2	0.61
Age (years)	59.1 ± 10.5	61.6 ± 11.7	60.0 ± 10.6	0.13
BMI (kg/m^2^)	24.3 ± 3.9	24.0 ± 3.4	24.1 ± 2.9	0.72
Diabetes duration (years)	10.7 ± 8.2	10.7 ± 8.8	10.8 ± 6.2	1.00
HbA1c (%)	8.4 ± 2.0	8.3 ± 2.0	8.1 ± 2.0	0.66
Systolic BP (mmHg)	139.5 ± 19.5	137.9 ± 21.3	143.4 ± 17.2	0.57
Diastolic BP (mmHg)	83.4 ± 11.4	81.8 ± 12.9	85.9 ± 9.7	0.31
Triglycerides (mmol/L)	1.7 ± 1.2	1.9 ± 2.3	1.8 ± 1.9	0.50
HDL-C (mmol/L)	1.4 ± 0.4	1.4 ± 0.4	1.4 ± 0.3	1.00
LDL-C (mmol/L)	3.2 ± 0.8	3.2 ± 0.9	3.1 ± 0.6	0.90
AST (IU/L)	24.8 ± 9.7	27.1 ± 14.2	25.2 ± 8.4	0.22
ALT (IU/L)	26.6 ± 16.4	30.2 ± 27.2	27.7 ± 11.6	0.31
GGT (IU/L)	36.6 ± 33.1	42.2 ± 36.8	43.6 ± 24.1	0.31
Log GGT (IU/L)	1.47 ± 0.30	1.51 ± 0.30	1.57 ± 0.26	0.08
Hypertension (%)	60.3	59.2	76.9	0.50
Dyslipidemia (%)	68.8	70.8	38.5	0.07
Ever smoker (%)	45.4	41.2	33.3	0.70
Alcohol intake (%)	51.4	46.7	38.5	0.52
Therapy components				
Hypoglycemic agents				
Oral hypoglycemic agents (%)	64.1	65.5	53.8	0.72
Insulin (%)	23.0	19.3	30.8	0.52
Antihypertensive agents				
ACE inhibitors or ARBs(%)	20.3	16.0	7.7	0.44
Others(%)	23.5	26.9	23.1	0.77
Agents for hyperlipidemia				
Fibrates(%)	0.9	0.8	7.7	0.15
Statins(%)	8.3	6.7	0.0	0.70
Others (%)	0.9	1.7	0.0	0.67

Data are percentages or mean ± standard deviation.

T2DM, type 2 diabetes mellitus; GGT, γ-glutamyltransferase; BMI, body mass index; HbA1c, hemoglobin A1c; BP, blood pressure; HDL-C, high-density lipoprotein cholesterol; LDL-C, low-density lipoprotein cholesterol; AST, aspartate aminotransferase; ALT, alanine aminotransferase; ACE, angiotensin converting enzyme; ARB, angiotensin II receptor blocker.

Among the general subjects, the mean log GGT values were also significantly higher in G allele carriers than in non-carriers (1.47 ± 0.29 IU/L vs. 1.43 ± 0.29 IU/L, *P* = 0.03). Factors associated with the serum GGT level stratified by the *GGT1* genotype in the general subjects are shown in Table 2. Fatty liver, dyslipidemia, ever-smoking, drinking, the ALDH2*2 allele and the BMI, transaminase, HbA1c, BP and triglyceride values were significantly associated with the GGT levels, irrespective of the *GGT1* genotype, while the fasting plasma glucose, HDL-C and LDL-C levels were significantly associated with the GGT levels only in G allele carriers. In the T2DM subjects, ever-smoking, drinking, the ALDH2*2 allele and the transaminase, diastolic BP and triglyceride levels were significantly associated with the GGT levels, irrespective of the *GGT1* genotype, while the HbA1c level was significantly associated with the GGT level only in non-carriers (Additional file 1: Table S1).

**Table 2:** **Factors associated with the serum GGT levels stratified by the** ***GGT1*** **genotype in the general subjects in the univariate regression model**

	**GGT (IU/L)**					
	***GGT1*** **A/A genotype**			***GGT1*** **A/G or G/G genotype**		
	**B**	**SE**	***P*** **-value**	**B**	**SE**	***P*** **-value**
Fatty liver	24.61	5.24	<0.001	20.67	5.16	<0.001
Dyslipidemia	11.08	4.34	0.01	15.16	4.73	0.001
Ever smoking	19.68	4.31	<0.001	16.66	4.72	<0.001
Drinking	18.13	4.30	<0.001	15.94	4.70	0.001
ALDH2*2 allele	−12.60	4.43	0.005	−5.90	4.80	0.22
BMI	3.76	0.70	<0.001	2.13	0.76	0.006
AST	3.24	0.17	<0.001	2.09	0.36	<0.001
ALT	1.67	0.14	<0.001	1.62	0.20	<0.001
HbA1c	8.07	1.81	<0.001	6.50	2.66	0.02
Systolic BP	0.27	0.13	0.04	0.35	0.15	0.02
Diastolic BP	0.84	0.20	<0.001	0.65	0.23	0.005
Triglycerides	0.16	0.03	<0.001	0.28	0.03	<0.001
Fasting plasma glucose	0.22	0.12	0.06	**0.53**	**0.15**	**0.001**
HDL-C	0.05	0.13	0.68	**−0.38**	**0.14**	**0.005**
LDL-C	0.001	0.09	0.99	**0.18**	**0.09**	**0.04**

Bold typeface indicates data that are significant only in the G allele carriers.

GGT, γ-glutamyltransferase; B, partial regression coefficient; SE; standard error; ALDH2, aldehyde dehydrogenase 2; BMI, body mass index; AST, aspartate aminotransferase; ALT, alanine aminotransferase; HbA1c, hemoglobin A1c; BP, blood pressure; HDL-C, high-density lipoprotein cholesterol; LDL-C, low-density lipoprotein cholesterol.

**Figure 1:**
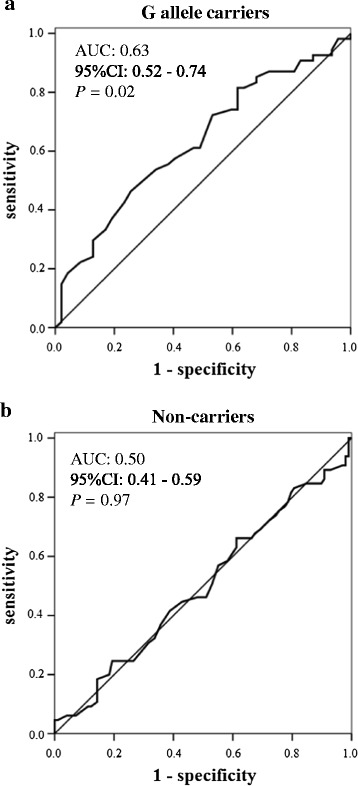
The ROC curves of HDL-C for high baPWV (≥1750 cm/sec) in the *GGT1 G* allele carriers **(a)** and non-carriers **(b)** in T2DM subjects. The HDL-C level at baseline was found to be a significant predictor of a high baPWV at the last point of follow-up only in G allele carriers. The optimal cut-off value for the HDL-C level for a high baPWV in G allele carriers was 1.1 mmol/L (sensitivity: 46.3%; specificity: 74.5%). AUC, area under the curve; CI, confidence interval; ROC, receiver operating characteristic; HDL-C, high-density lipoprotein cholesterol; baPWV, brachial-ankle pulse wave velocity; T2DM, type 2 diabetes mellitus.

The *GGT1* G allele was significantly associated with the risk of a high baPWV (≥1,750 cm/sec) in the T2DM subjects (OR 1.80, *P* = 0.008), in addition to age, hypertension, the diabetes duration, low HDL-C (<1.0 mmol/L) and drinking in the univariate models (Table 3). According to the multiple regression models, age and hypertension were found to be independent risk factors for a high baPWV irrespective of the *GGT1* genotype (Table 4)(Additional file 1: Table S2). A significant interactive effect of the *GGT1* genotype and the HDL-C level on the risk of a high baPWV was observed in the generalized estimating equations approach (*P* = 0.04). Notably, the risk of a high baPWV was significantly greater in G allele carriers with a low HDL-C level (OR 2.49, *P* = 0.04) than in non-carriers with a normal HDL-C level (Table 5). The baPWV values were also higher in *GGT1* G allele carriers with a low HDL-C level than in non-carriers with a normal HDL-C level (B: 150.84 cm/sec, *P* = 0.06). Moreover, the HDL-C level at baseline was found to be a significant predictor of a high baPWV at the last point of follow-up only in G allele carriers according to the ROC curve analysis (AUC 0.63, 95%CI 0.52 - 0.74, *P* = 0.02) (Figure 1a and b). The optimal cut-off value for the HDL-C level for predicting a high baPWV in G allele carriers was 1.1 mmol/L (sensitivity: 46.3%; specificity: 74.5%).

**Table 3:** **Association between a high baPWV (≥1,750 cm/sec) and the covariates in the T2DM subjects**

	**High baPWV**			
	**Univariate**		**Multivariate**	
	**OR (95% CI)**	***P*** **-value**	**OR (95% CI)**	***P*** **-value**
*GGT1* G allele	**1.80 (1.16 - 2.78)**	**0.008**	1.54 (0.97 - 2.43)	0.07
Age	**1.11 (1.08 - 1.14)**	**< 0.001**	**1.10 (1.07 - 1.13)**	**< 0.001**
Hypertension	**3.84 (2.47 - 5.98)**	**< 0.001**	**3.37 (2.11 - 5.38)**	**< 0.001**
Diabetes duration	**1.06 (1.03 - 1.08)**	**< 0.001**	1.02 (0.997 - 1.05)	0.09
HDL-C < 1.0 mmol/L	**1.71 (1.07 - 2.74)**	**0.03**	1.40 (0.87 - 2.26)	0.16
Drinking	**0.63 (0.42 - 0.94)**	**0.02**	1.06 (0.66 - 1.69)	0.83
ALT	0.99 (0.98 - 1.00)	0.07	1.01 (0.99 - 1.02)	0.40
ALDH2*2 allele	1.43 (0.94 - 2.20)	0.099	1.47 (0.90 - 2.40)	0.13
GGT	1.00 (0.995 - 1.01)	0.60		
Female	1.18 (0.75 - 1.85)	0.49		
BMI	0.99 (0.93 - 1.05)	0.76		
HbA1c	0.94 (0.84 - 1.05)	0.26		
AST	1.01 (0.99 - 1.03)	0.52		
LDL-C ≥ 3.6 mmol/L	0.84 (0.57 - 1.23)	0.37		
Triglycerides ≥ 1.7 mmol/L	1.14 (0.77 - 1.69)	0.51		

Bold typeface indicates data that are statistically significant. baPWV, brachial-ankle pulse wave velocity; T2DM, type 2 diabetes mellitus; OR, odds ratio; CI, confidence interval; GGT, γ-glutamyltransferase; HDL-C, high-density lipoprotein cholesterol; ALT, alanine aminotransferase; ALDH2, aldehyde dehydrogenase 2; BMI, body mass index; HbA1c, hemoglobin A1c; AST, aspartate aminotransferase; LDL-C, low-density lipoprotein cholesterol.

**Table 4:** **Interactive effects between the** ***GGT1*** **genotype and serum HDL-C levels on the baPWV in the T2DM subjects**

**HDL-C**	***GGT1*** **genotype**	**High baPWV** ^*^		**baPWV (cm/sec)**		
		**OR** ^**†**^ **(95% CI)**	***P*** **-value**	**B** ^**†**^	**SE**	***P*** **-value**
≥ 1.0 mmol/L	A/A genotype	1	-	0	-	-
	A/G or G/G genotype	1.49 (0.91 - 2.44)	0.11	47.38	38.60	0.22
<1.0 mmol/L	A/A genotype	1.31 (0.73 - 2.35)	0.37	58.32	54.50	0.29
	A/G or G/G genotype	**2.49 (1.07 - 5.82)**	**0.04**	150.84	79.19	0.06

Bold typeface indicates data that are statistically significant.

^*^baPWV ≥ 1750 cm/sec.

^†^Adjusted by age, hypertension, diabetes duration, drinking, ALT level and ALDH2*2 allele.

GGT, γ-glutamyltransferase; HDL-C, high-density lipoprotein cholesterol; baPWV, brachial-ankle pulse wave velocity; T2DM, type 2 diabetes mellitus; OR, odds ratio; CI, confidence interval; B, partial regression coefficient; SE, standard error.

**Table 5:** **Association between the risk of DR and the covariates stratified by the** ***GGT1*** **genotype in the T2DM subjects in the multiple regression model**

	***GGT1*** **A/A genotype**		***GGT1*** **A/G or G/G genotype**	
	**HR (95% CI)**	***P*** **-value**	**HR (95% CI)**	***P*** **-value**
HbA1c	**1.46 (1.23 - 1.73)**	**<0.001**	1.17 (0.87 - 1.58)	0.30
Female	**3.49 (1.25 - 9.72)**	**0.02**	3.13 (0.74 - 13.33)	0.12
Age	**0.94 (0.90 - 0.98)**	**0.005**	1.02 (0.96 - 1.09)	0.49
ALDH2*2 allele	2.21 (0.99 - 4.91)	0.052	**7.88 (1.53 - 40.54)**	**0.014**
HDL-C	1.01 (0.99 - 1.04)	0.35	**0.94 (0.90 - 0.99)**	**0.02**
Diabetes duration	1.04 (0.97 - 1.11)	0.26	**1.11 (1.01 - 1.23)**	**0.03**
GGT	1.00 (0.98 - 1.01)	0.50	1.02 (0.999 - 1.03)	0.07
Hypertension	1.90 (0.74 - 4.90)	0.18	1.86 (0.46 - 7.61)	0.39
Drinking	1.45 (0.54 - 3.89)	0.46	1.89 (0.41 - 8.73)	0.42
ALT	1.00 (0.97 - 1.03)	0.89	0.99 (0.97 - 1.02)	0.56

Bold typeface indicates data that are statistically significant. DR, diabetic retinopathy; GGT, γ-glutamyltransferase; T2DM, type 2 diabetes mellitus; HR, hazard ratio; CI, confidence interval; HbA1c, hemoglobin A1c; ALDH2, aldehyde dehydrogenase 2; HDL-C, high-density lipoprotein cholesterol; ALT, alanine aminotransferase.

In the general subjects, a significant interactive effect of the *GGT1* genotype and dyslipidemia on the baPWV values was observed (*P* = 0.02). The mean baPWV values in G allele carriers were significantly higher among the subjects with dyslipidemia than those without dyslipidemia (1,790.8 ± 370.4 cm/sec vs. 1,617.8 ± 347.8 cm/sec, *P* = 0.01), while the values did not differ in non-carriers. Similarly, the mean baPWV values in G allele carriers were higher among the subjects with a low HDL-C level than those with a normal HDL-C level, although the difference did not reach statistical significance (1,988.3 ± 381.4 cm/sec vs. 1,705.1 ± 367.8 cm/sec, *P* = 0.19) (Additional file 1: Table S3). The HDL-C level was also significantly associated with a high baPWV only in G allele carriers among the general subjects (AUC 0.63, 95%CI 0.52 - 0.74, *P* = 0.02) (Additional file 2: Figure S1a and b).

Forty-five (21.0%) patients were newly diagnosed with DR during the observation period. The HbA1c level was found to be the only risk factor for the incidence of DR in the univariate regression model. When stratified by the *GGT1* genotype, the HbA1c level, a female gender and age were identified to be significantly associated with the incidence of DR in non-carriers, whereas the diabetes duration, ALDH2*2 allele and HDL-C level were significantly associated with the incidence of DR in G allele carriers (Table 5). A significant interactive effect of the *GGT1* genotype and HDL-C level on the risk of DR was also observed (*P* = 0.02).

## Discussion

The novel findings of this study are as follows. First, a *GGT1* variant, rs4820599, the G allele and a low HDL-C level were identified to be risk factors for a high baPWV in Japanese subjects with T2DM in the longitudinal analysis. Interestingly, a significant interactive effect of the *GGT1* genotype and low HDL-C level on the risks of both a high baPWV and DR was found. Second, the HDL-C level at baseline was identified to be a significant predictor of a high baPWV only in G allele carriers according to the ROC analysis. This result in the T2DM patients was also noted in the general population. Third, the *GGT1* genotype was not associated with the risk of DR, although it affected the principal factors involved in the risk of DR. Fourth, the associations between the levels of GGT and fasting plasma glucose, HDL-C and LDL-C and between dyslipidemia and a high baPWV were significant only in G allele carriers among the general subjects. In this study, the triglyceride levels were positively associated with the serum GGT levels but not the risk of a high baPWV or DR, and the LDL-C levels were positively associated with the serum GGT levels only in the general subjects with the G allele.

The mean serum log GGT level was significantly higher in G allele carriers than in non-carriers among both the T2DM patients and the general population. The expression of GGT increases as an adaptive response to oxidative stress, although the mechanism underlying GGT regulation in response to oxidative stress remains unclear [9,32]. Human *GGT* is a multigene family consisting of at least seven *GGT* genes or pseudogenes, in which a major *GGT1* and minor *GGT5* have been found to encode proteins exhibiting GGT activity [32]. A variant, rs4820599, on the *GGT1* gene is predicted to be located at a transcription factor binding site, which can result in differential gene transcription, according to FuncPred (http://snpinfo.niehs.nih.gov/snpinfo/snpfunc.htm, accessed 3/31/2015). This fact may explain the findings of the present study and previous observations obtained using GWAS [27-29]. In addition to *GGT1*, however, population-based GWAS have revealed evidence of an association between the serum GGT level and a series of SNPs in candidate genes, *i.e. HNF1A* in various ethnic groups [27-29], *C14orf73* and *RORA* in subjects of European descent [29], *MYL2, C12olf51* and *OAS1* in individuals of East Asian descent [28] and *ALDH2* in Japanese [33]. A pathway analysis of the SNP associations showed significant overlap between genes affecting the GGT level and those affecting common metabolic and inflammatory diseases under the control of the Hepatic Nuclear family [29]. Therefore, our results provide only the first glimpse into the association between one of these SNPs and the GGT level, factors affecting the GGT level and the risk of vascular disease resulting from metabolic and inflammatory diseases, as represented by T2DM.

The baPWV is used in epidemiological and clinical research as an index of arterial stiffness and atherosclerosis and is indicated to be an independent predictor of CVD [30,34-36]. A few epidemiological studies have examined the association between the GGT level and baPWV, although the findings are inconsistent [35,36]. In the present study, the *GGT1* G allele was identified to be a risk factor for a high baPWV, while the GGT level was found to be a risk factor in non-carriers. GGT is expressed on the cell surface membrane of most cell types, although only liver GGT is detected in the serum, and the relationship between the cellular GGT and serum GGT expressions is unknown [6,37]. The serum GGT level appears to be associated with the risk of CVD from the early stage of arterial stiffness through the onset of CVD events [2,4,6,7,35,36,38]. This is because oxidative stress, vascular inflammation and endothelial dysfunction play a central role in the pathogenesis of atherosclerosis [39,40], and because the serum GGT level is proposed to be an early and sensitive marker of oxidative stress [7,9,32]. A key question is whether the serum GGT level is an simply innocent marker or whether its activity is causally involved in the development of arterial stiffness and atherogenesis, as shown in atheroma plaques [6,7]. Cellular GGT is known to be involved in the generation of reactive oxygen species in the presence of transition metals and serum GGT is speculated to promote the oxidation of circulating lipoproteins [6,7,9,41].

The serum HDL-C level has been reported to be inversely related to the baPWV in a general Chinese population (50 to 90 years of age) and the aortic PWV in other populations [42]. An isolated low HDL-C level is a dyslipidemic phenotype that appears to be more prevalent among Asian populations, in whom low HDL-C levels are strongly associated with an increased CHD risk [43]. Several potential mechanisms may explain the association between the serum HDL-C level and CVD [15,20-22,42-44]. HDL has a role in reverse cholesterol transport and other direct actions on numerous cell types, thereby reducing cardiovascular risks. In endothelial cells and their progenitors, HDL prevents apoptosis and stimulates proliferation and migration. HDL also has diverse anti-inflammatory actions in both endothelial cells and leukocytes. In vascular smooth muscles, HDL attenuates proinflammatory, promigratory and degradative processes. HDL has an antithrombotic effect via its actions on the endothelium and platelets. In addition, HDL protects pancreatic β-cells from apoptosis, decreases the white adipose tissue mass, increases energy expenditures and promotes the production of adiponectin, which possesses its own vascular protective properties. However, the classic HDL (concentration) hypothesis is gradually being replaced by the triglyceride hypothesis and the HDL function hypothesis due to the failure of clinical trials and negative results for human genetics [12-14,18-22]. The classic entity of ‘diabetic dyslipidemia’ is characterized by the so-called atherogenic lipid triad, consisting of an increase in small dense LDL particles and triglyceride-rich lipoproteins and a decrease in HDL-C [12-17,22]. In this study, the triglyceride levels were positively associated with the serum GGT levels, but not any of the risk factors for a high baPWV or DR in both the T2DM patients and general subjects. In order to prove that a low HDL-C level, rather than a high triglyceride level, is a risk factor for a high PWV, we applied a multivariate model including triglycerides on purpose despite the insignificance of this parameter as a covariate. Most serum GGT is bound to lipoproteins, more dominantly to Apo A than to Apo B, although the ratio may change depending on the serum lipid profile [6,8-11,41]. All things considered, the serum GGT level is associated with cardiovascular risks, including dyslipidemia, more greatly in G allele carriers than in non-carriers, and coexisting low HDL-C may lead to increased LDL-associated GGT and GGT-dependent LDL oxidation followed by the possible development of significant arterial stiffness in this population.

The interactive effect of the *GGT1* genotype and a low HDL-C level on the risk of DR was also significant in the current study, and a low HDL-C level was found to be an independent risk factor for DR only in G allele carriers. Oxidative stress plays a pivotal role in the development of DR as well as macrovascular diseases [45-49]. The metabolic abnormalities associated with diabetes cause mitochondrial superoxide overproduction in the endothelial cells of both small and large vessels [45,48,49]. This increased superoxide production causes the activation of major pathways involved in the pathogenesis of various complications, such as polyol pathway flux as well as the increased formation of advanced glycation end products, activation of protein kinase C and over-activity of the hexosamine pathway [45,48,49]. The overexpression of superoxide dismutase in transgenic diabetic mice prevents DR, nephropathy and cardiomyopathy [48,49]. In addition to two principal and reversible risk factors for DR, blood glucose and BP, a low HDL-C level is indicated to be a risk factor for DR [16,23,25,46,49]. Many of the pathogenic effects of lipoproteins occur after these particles leak from the circulation [16,25,49]. Du *et al.* [25] identified extravasated Apo A1 and Apo B in human diabetic eyes and showed that native HDL completely blocks oxidative stress and the apoptosis of retinal pigment epithelial cells induced by heavily oxidized glycated LDL, thereby suggesting an important new role for extravasated and modified plasma lipoproteins in promoting DR. We speculate that GGT may co-localize with extravasated HDL and LDL and possibly take part in the pathogenesis of DR in G allele carriers with a low HDL-C level. In the present study, among G allele carriers, the diabetes duration and ALDH2*2 allele were other independent risk factors for DR. We previously reported a significant association between the ALDH2*2 allele and DR, as follows: the incidence of DR is significantly higher in ALDH2*2 allele carriers with a high GGT level (>37 IU/L for males and > 26 IU/L for females) than in non-carriers with a high or low GGT level; a high GGT level in non-carriers is most significantly associated with drinking habits, while that in ALDH2*2 allele carriers is significantly associated with multiple cardiovascular risk factors; and ALDH2 therefore may protect the vasculature against reactive aldehydes generated under conditions of sustained oxidative stress [50]. In non-carriers, a female gender, age and the HbA1c level, which is associated with a high GGT level, were found to be independent risk factors for DR.

Diabetes and hypertension promote adverse changes throughout the vascular tree, eliciting both macrovascular and microvascular complications [34,38,45,46]. Increased arterial stiffness and microvascular remodeling are the most prevalent and earliest forms of organ damage in these diseases [34,38,45,46]. Therefore, diabetic subjects with a high PWV and/or MVCs appear to be particularly prone to developing accelerated atherosclerosis and premature death [38,46,47]. A significant positive association between the presence of DR and baPWV has been reported in Japanese T2DM patients without macrovascular complications [34]. The association between a high baPWV and DR is likely to exist in G allele carriers, although this finding did not reach statistical significance in the present study due to the small sample size (data not shown). Although fibrates fail to reduce cardiovascular events in patients treated with statins in general [14,18], fenofibrate has received major attention as a novel medical treatment for DR and other diabetes-induced MCVs [24]. This is because well-designed clinical trials, *i.e.* the Fenofibrate Intervention and Event Lowering in Diabetes (FIELD) and ACCORD trials, demonstrated large reductions in the progression of DR and the subsequent need for laser intervention, in addition to reductions in adverse renal and neurological outcomes, in patients with T2DM [24]. Fenofibrate regulates the expression of many different genes, with a range of beneficial effects for lipid control, inflammation, angiogenesis and cell apoptosis [24,51]. Interestingly, treatment with 200 mg/day of fenofibrate for 48 weeks in patients with non-alcoholic fatty liver disease was recently shown to improve metabolic syndrome, in addition to glucose and liver parameters, including a reduction in the serum GGT level of 39% [51]. These findings and the current results suggest that the early detection and treatment of low HDL-C levels in populations at high risk for MVC and CVD, such as T2DM patients with arterial stiffness and/or DR, may be an attractive therapeutic target for prevention [6,7,15-17,24,34,38,45,46,51]. The *GGT1* G allele is a novel candidate risk factor in this population and we hope to reanalyze the findings of previous clinical trials of agents used to increase the HDL-C levels, stratifying the outcome according to the *GGT1* genotype.

The strength of this study is that significant interactive effects between the *GGT1* genotype and a low HDL-C level were observed on both a high baPWV and DR in two different populations and different analyses. The crucial limitations of this study include the retrospective study design, small sample size and lack of information on factors such as the effects of drug therapy, the HDL-C fraction and function and specific biomarkers of oxidative stress.

We herein presented for the first time the significant interactive effects of the *GGT1* G allele and a low HDL-C level on a high baPWV and DR. These findings may suggest a common pathological mechanism in case of diabetic macro- and micro-angiopathy and prompt repeat-analyses of previous trials according to the *GGT1* genotype as well as the development of future clinical trials comparing the efficacy of agents increasing the HDL-C level in improving angiopathy between *GGT1* genotypes. However, well-designed studies in larger cohorts are still needed to further confirm our results.

## Additional files

Additional file 1: Table S1. Factors associated with serum GGT level stratified by the *GGT1* genotype in the T2DM subjects in the univariate regression model. **Table S2.** Interactive effects between the *GGT1* genotype and dyslipidemia on the baPWV levels in the general subjects. Additional file 2: Figure S1. The ROC curves of HDL-C for high baPWV (≥1750 cm/sec) in the *GGT1 G* allele carriers (a) and non-carriers (b) in the general subjects. The HDL-C level was associated significantly with a high baPWV only in G allele carriers (AUC 0.63, 95%CI 0.52 - 0.74, *P* = 0.02).AUC, area under the curve; CI, confidence interval; ROC, receiver operating characteristic; HDL-C, high-density lipoprotein cholesterol; baPWV, brachial-ankle pulse wave velocity.

## Abbreviations

ACCORD: Action to Control Cardiovascular Risk in Diabetes
ACE: Angiotensin converting enzyme
ALDH2: Aldehyde dehydrogenase 2
ALT: Alanine aminotransferase
Apo: Apolipoprotein
ARB: Angiotensin II receptor blocker
AST: Aspartate aminotransferase
AUC: Area under the curve
B: Partial regression coefficient
baPWV: brachial-ankle pulse wave velocity
BMI: Body mass index
BP: Blood pressure
CAD: Coronary artery disease
CI: Confidence interval
CVD: Cardiovascular disease
DR: Diabetic retinopathy
FIELD: Fenofibrate Intervention and Event Lowering in Diabetes
GGT: γ-glutamyltransferase
GWAS: Genome-wide association study
HbA1c: Hemoglobin A1c
HDL-C: High-density lipoprotein cholesterol
HF: Heart failure
HR: hazard ratio
LDL-C: Low-density lipoprotein cholesterol
MVC: Microvascular complication
NPDR: Nonproliferative diabetic retinopathy
OR: Odds ratio
PDR: Proliferative diabetic retinopathy
ROC: Receiver operating characteristic
SNP: Single-nucleotide polymorphism
T2DM: Type 2 diabetes mellitus
