# Supplementary material for: Interactive effects of a common γ-glutamyltransferase 1 variant and low high-density lipoprotein-cholesterol on diabetic macro- and micro-angiopathy
Source: Cardiovasc Diabetol. 2015 May 8;14:49. doi: 10.1186/s12933-015-0212-5 (PMC4428095; doi:10.1186/s12933-015-0212-5)
Supplement: Additional file 1: Table S1. — Factors associated with serum GGT level stratified by the GGT1 genotype in the T2DM subjects in the univariate regression model. Table S2. Interactive effects between the GGT1 genotype and dyslipidemia on the baPWV levels in the general subjects. [file 12933_2015_212_MOESM1_ESM.docx]

Table S1. Factors associated with serum GGT level stratified by the *GGT1* genotype in the T2DM

subjects in the univariate regression model

|  | GGT (IU/L) | | | | | | |
| --- | --- | --- | --- | --- | --- | --- | --- |
|  | *GGT1* A/A genotype | | |  | *GGT1* A/G or G/G genotype | | |
|  | B | SE | *P*-value |  | B | SE | *P*-value |
| Dyslipidemia | 8.41 | 4.83 | 0.08 |  | 7.86 | 6.61 | 0.24 |
| Ever smoking | 20.76 | 4.33 | < 0.001 |  | 26.10 | 5.96 | < 0.001 |
| Drinking | 13.73 | 4.41 | < 0.001 |  | 20.48 | 5.98 | 0.001 |
| *ALDH2*2* allele | -9.78 | 4.52 | 0.03 |  | -18.33 | 6.06 | 0.003 |
| BMI | 1.04 | 0.57 | 0.07 |  | 1.51 | 0.91 | 0.10 |
| AST | 1.34 | 0.21 | < 0.001 |  | 0.98 | 0.21 | < 0.001 |
| ALT | 0.76 | 0.13 | < 0.001 |  | 0.60 | 0.11 | < 0.001 |
| HbA1c | **2.21** | **1.12** | **0.049** |  | 0.40 | 1.55 | 0.80 |
| Systolic BP | 0.10 | 0.12 | 0.41 |  | 0.24 | 0.15 | 0.10 |
| Diastolic BP | 0.51 | 0.20 | 0.01 |  | 0.66 | 0.24 | 0.007 |
| Triglycerides | 0.08 | 0.02 | < 0.001 |  | 0.05 | 0.02 | 0.001 |
| HDL-C | -0.19 | 0.14 | 0.19 |  | -0.18 | 0.21 | 0.39 |
| LDL-C | 0.07 | 0.07 | 0.34 |  | -0.14 | 0.09 | 0.13 |

Bold typeface indicates data that are significant only in the A/A genotype.

Fatty liver was not included in this analysis, because of the deficit in data.

GGT, γ-glutamyltransferase; T2DM, type 2 diabetes mellitus diabetes mellitus; B, partial

regression coefficient; SE; standard error; ALDH2, aldehyde dehydrogenase 2; BMI, body mass index;

AST, aspartate aminotransferase; ALT, alanine aminotransferase; ; HbA1c, hemoglobin A1c; BP, blood

pressure; HDL-C, high-density lipoprotein cholesterol; LDL-C, low-density lipoprotein cholesterol.

Table S2. Association between a high baPWV (≥ 1,750 cm/sec) and the covariates stratified by the *GGT1* genotype in the T2DM subjects

|  | High baPWV | | | | | | | | | | | |
| --- | --- | --- | --- | --- | --- | --- | --- | --- | --- | --- | --- | --- |
|  | *GGT1* A/A genotype | | | | |  | *GGT1* A/G or G/G genotype | | | | |  |
|  | Univariate | |  | Multivariate | |  | Univariate | |  | Multivariate | |  |
|  | OR (95% CI) | *P*-value |  | OR (95% CI) | *P*-value |  | OR (95% CI) | *P*-value |  | OR (95% CI) | *P*-value |  |
| Age | **1.09 (1.06 - 1.12)** | **< 0.001** |  | **1.09 (1.05 - 1.13)** | **< 0.001** |  | **1.14 (1.08 - 1.19)** | **< 0.001** |  | **1.13 (1.07 - 1.19)** | **< 0.001** |  |
| Hypertension | **2.96 (1.69 - 5.16)** | **< 0.001** |  | **2.31 (1.30 - 4.10)** | **0.004** |  | **5.65 (2.75 - 11.58)** | **< 0.001** |  | **5.47 (2.58 - 11.60)** | **< 0.001** |  |
| Diabetes duration | **1.05 (1.02 - 1.07)** | **0.001** |  | 1.02 (0.99 - 1.05) | 0.13 |  | **1.08 (1.03 - 1.13)** | **0.001** |  | 1.02 (0.98 - 1.06) | 0.42 |  |
| HDL-C < 1.0 mmol/L | 1.57 (0.89 - 2.77) | 0.12 |  |  |  |  | **2.74 (1.09 - 6.91)** | **0.03** |  | 1.77 (0.71 - 4.42) | 0.22 |  |
| Drinking | 0.71 (0.43 - 1.18) | 0.19 |  |  |  |  | 0.56 (0.29 - 1.06) | 0.08 |  | 1.02 (0.50 - 2.07) | 0.97 |  |
| ALT | 1.00 (0.98 - 1.02) | 0.95 |  |  |  |  | **0.97 (0.94 - 0.998)** | **0.04** |  | 1.00 (0.97 - 1.02) | 0.76 |  |
| *ALDH2*2* allele | 1.44 (0.85 - 2.46) | 0.18 |  |  |  |  | 1.52 (0.76 - 3.03) | 0.23 |  |  |  |  |
| GGT | **1.01 (1.00 - 1.02)** | **0.048** |  | **1.02 (1.01 - 1.03)** | **0.004** |  | 0.99 (0.98 - 1.01) | 0.26 |  |  |  |  |
| Female | 1.21 (0.69 - 2.15) | 0.51 |  |  |  |  | 1.12 (0.53 - 2.34) | 0.77 |  |  |  |  |
| BMI | 1.02 (0.95 - 1.09) | 0.61 |  |  |  |  | 0.95 (0.86 - 1.06) | 0.35 |  |  |  |  |
| HbA1c | 0.89 (0.77 - 1.04) | 0.13 |  |  |  |  | 1.01 (0.83 - 1.22) | 0.93 |  |  |  |  |
| AST | 1.01 (0.99 - 1.04) | 0.24 |  |  |  |  | 1.00 (0.97 - 1.03) | 0.98 |  |  |  |  |
| LDL-C ≥ 3.6 mmol/L | 0.81 (0.50 - 1.31) | 0.39 |  |  |  |  | 0.82 (0.44 - 1.54) | 0.54 |  |  |  |  |
| Triglycerides ≥ 1.7 mmol/L | 1.33 (0.82 - 2.16) | 0.25 |  |  |  |  | 0.99 (0.52 - 1.88) | 0.97 |  |  |  |  |

baPWV, brachial-ankle pulse wave velocity; T2DM, type 2 diabetes mellitus; OR, odds ratio; CI, confidence interval;

GGT, γ-glutamyltransferase; HDL-C, high-density lipoprotein cholesterol; ALT, alanine aminotransferase;

ALDH2, aldehyde dehydrogenase 2; BMI, body mass index; HbA1c, hemoglobin A1c; AST, aspartate aminotransferase;

LDL-C, low-density lipoprotein cholesterol

Table S3. Interactive effects between the *GGT1* genotype and dyslipidemia on the baPWV levels in the

general subjects

|  | baPWV (cm/sec) | | | |  |
| --- | --- | --- | --- | --- | --- |
| *GGT1* genotype | n | Non-dyslipidemia | n | Dyslipidemia | *P*-value |
| A/A genotype | 69 | 1,709.0 ± 336.0 | 78 | 1,688.8 ± 332.4 | 0.72 |
| A/G or G/G genotype | 55 | **1,617.8 ± 347.8** | 66 | **1,790.8 ± 370.4** | **0.01** |
| *GGT1* genotype | n | HDL-C ≥ 1.0 mmol/L | n | HDL-C < 1.0 mmol/L | *P*-value |
| A/A genotype | 144 | 1,701.4 ± 334.8 | 3 | 1,551.7 ± 232.2 | 0.44 |
| A/G or G/G genotype | 118 | 1,705.1 ± 367.8 | 3 | 1,988.3 ± 381.4 | 0.19 |

The data are the means ± standard deviation.

GGT, γ-glutamyltransferase; baPWV, brachial-ankle pulse wave velocity; HDL-C, high-density lipoprotein

cholesterol.
