# Supplementary figures and images for: Interactive effects of a common γ-glutamyltransferase 1 variant and low high-density lipoprotein-cholesterol on diabetic macro- and micro-angiopathy
Source: Cardiovasc Diabetol. 2015 May 8;14:49. doi: 10.1186/s12933-015-0212-5 (PMC4428095; doi:10.1186/s12933-015-0212-5)

**a**

## G allele carriers

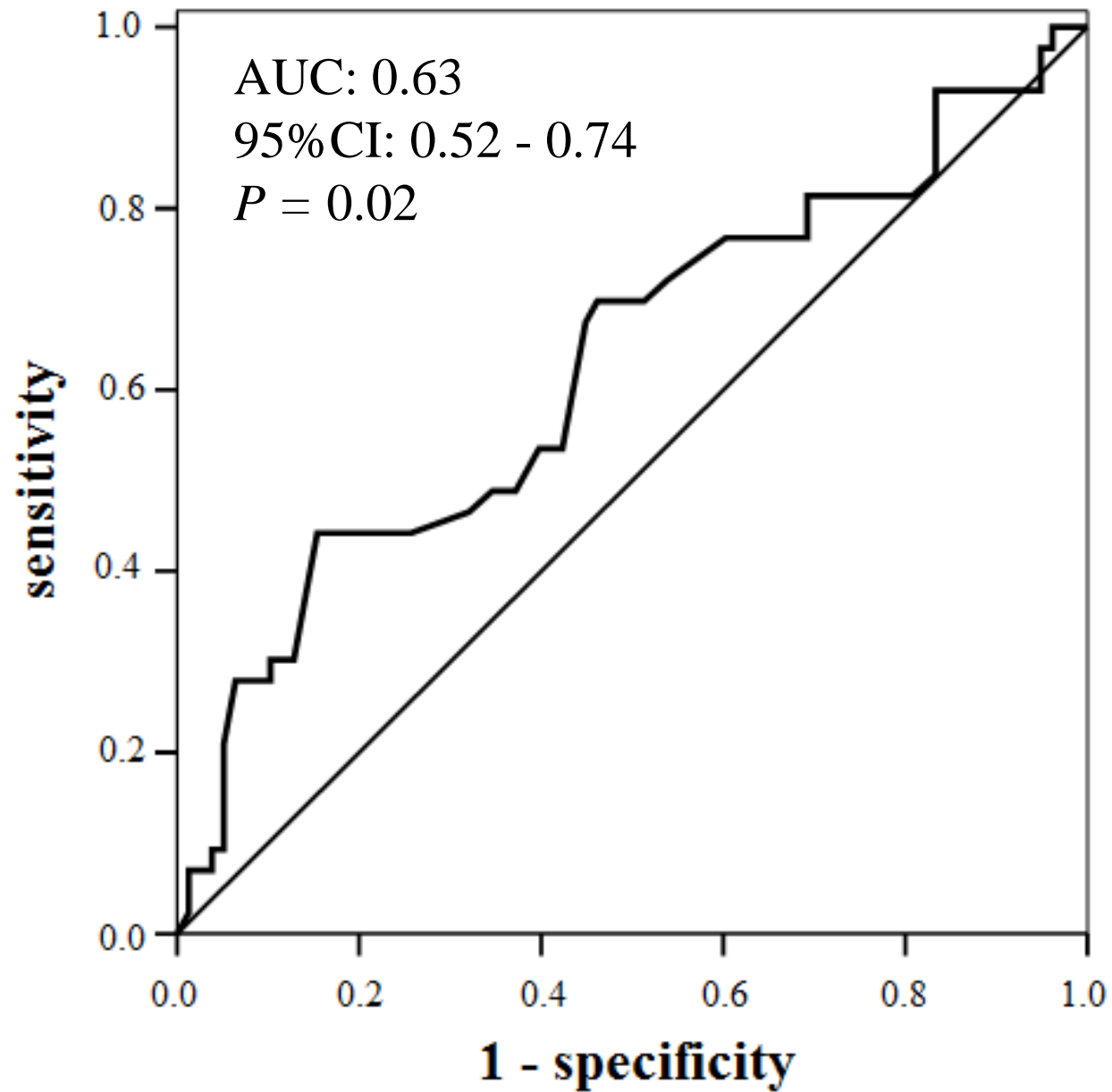

**b**

## Non-carriers

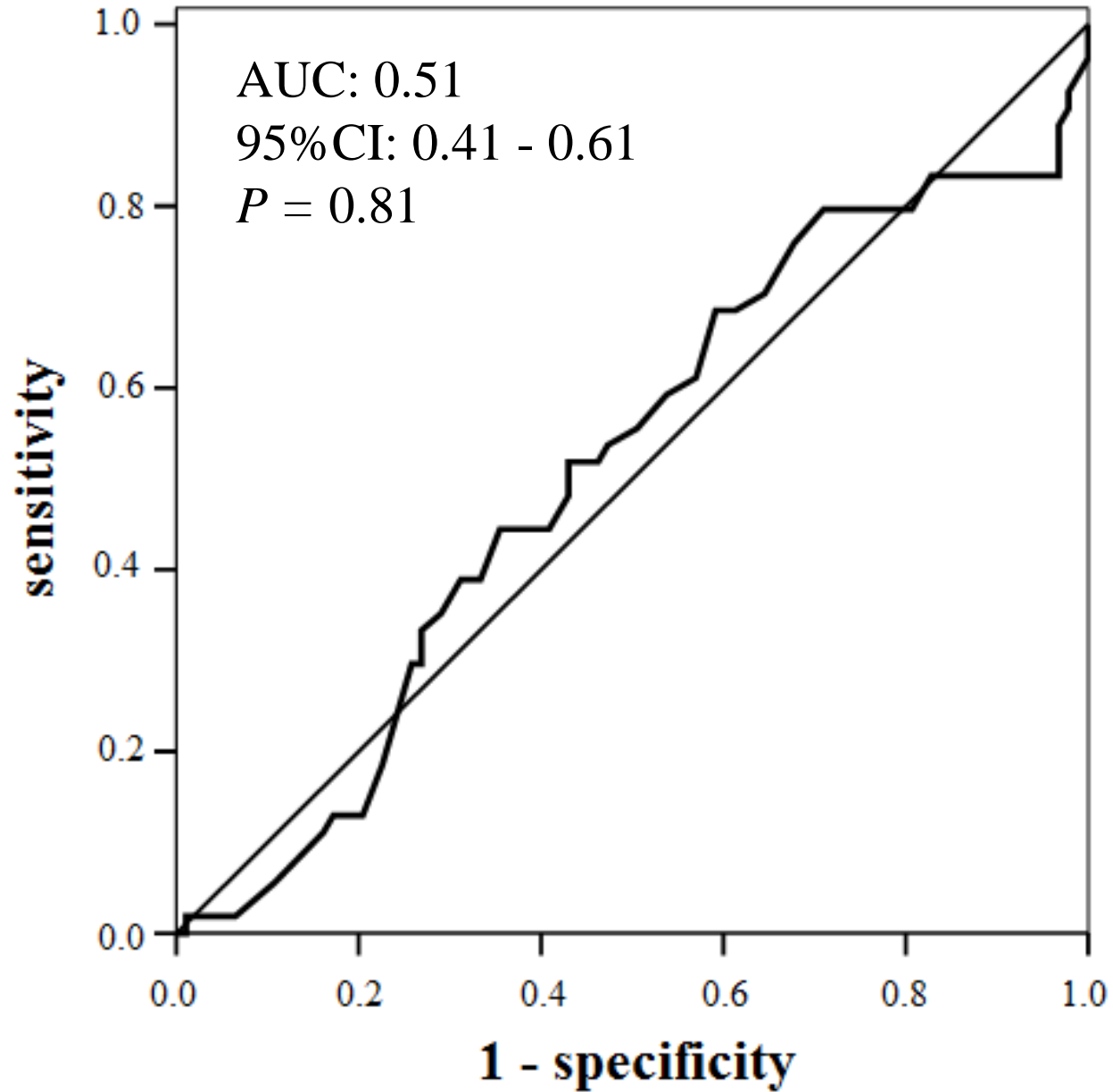

Supplement: Additional file 2: Figure S1. — The ROC curves of HDL-C for high baPWV (≥1750 cm/sec) in the GGT1 G allele carriers (a) and non-carriers (b) in the general subjects. The HDL-C level was associated significantly with a high baPWV only in G allele carriers (AUC 0.63, 95%CI 0.52 - 0.74, P = 0.02).AUC, area under the curve; CI, confidence interval; ROC, receiver operating characteristic; HDL-C, high-density lipoprotein cholesterol; baPWV, brachial-ankle pulse wave velocity. [file 12933_2015_212_MOESM2_ESM.pdf]
